# Unprotected sex among men who have sex with men living with HIV in Brazil: a cross-sectional study in Rio de Janeiro

**DOI:** 10.1186/1471-2458-14-379

**Published:** 2014-04-17

**Authors:** Cynthia Braga Cunha, Raquel Brandini De Boni, Maria Regina Cotrim Guimarães, Carolyn Yanavich, Valdilea Gonçalves Veloso, Ronaldo Ismerio Moreira, Brenda Hoagland, Beatriz Grinsztejn, Ruth Khalili Friedman

**Affiliations:** 1 Laboratório de Pesquisa Clinica em DST/AIDS, Fundação Oswaldo Cruz – Instituto de Pesquisa Clínica Evandro Chagas/IPEC, Avenue Brasil, 4365 – Manguinhos, Rio de Janeiro RJ CEP 21040-900, Brasil

**Keywords:** Men who have sex with men, HIV/AIDS, Unprotected anal intercourse, Low and middle income countries

## Abstract

**Background:**

Many countries are facing concentrated HIV epidemics among vulnerable populations, including men who have sex with men (MSM). Unprotected anal intercourse (UAI) is the main HIV transmission route among them and its understanding in the different cultures and how it relates to HIV transmission, re-infection and development of HIV antiretroviral resistance has important public health implications. Data on UAI among Brazilian MSM are scarce. This study aims to evaluate the prevalence and associated factors of UAI among HIV-infected MSM who had sex with seronegative or male partners with an unknown serostatus.

**Method:**

A cross-sectional study nested in a cohort was conducted in Rio de Janeiro, Brazil. The one hundred and fifty five MSM included in the study answered an ACASI interview and provided biological samples. Generalized linear models were used to identify variables associated with UAI.

**Results:**

Overall, UAI with an HIV-negative or unknown serostatus male partner was reported by 40.6% (63/155) of MSM. Lifetime sexual abuse or domestic violence was reported by 35.9%, being more frequent among MSM who reported UAI compared to those who did not (*P* = 0.001). Use of stimulants before sex was reported by 20% of the MSM, being slightly higher among those who reported UAI (27.0% vs. 15.2%; *P* = 0.072). Commercial sex was frequent among all MSM (48.4%). After multivariate modeling, the report of sexual abuse or domestic violence (OR = 2.70; 95% CI: 1.08-7.01), commercial sex (OR = 2.28; 95% CI: 1.04- 5.10), the number of male sexual partners (p = 0.039) and exclusively receptive anal intercourse (OR = 0.21; 95% CI: 0.06-0.75) remained associated with UAI. CD4 levels, HIV viral load and antiretroviral therapy were not associated with UAI.

**Conclusion:**

The UAI prevalence found with negative or unknown HIV status partners points out that other interventions are needed as additional prevention tools to vulnerable MSM. The main factors associated with UAI were a lifetime history of violence, commercial sex and the number of male sexual partners. This clustering of different behavioral, health and social problems in this population reinforce the need of a comprehensive approach on treating and preventing HIV among MSM.

## Background

Men who have sex with men (MSM) remain a vulnerable population for HIV infection across the world [1]. In Brazil, HIV prevalence among the general population is below 0.6% [2]. However, in the largest study conducted in the country, HIV prevalence among MSM ranged from 9.1% to 16% [3]. Soon after the onset of the HIV epidemic, there were reductions in transmission among MSM due to the expansion of condom use [4] or even sexual abstinence [5,6]. Although condoms represent an effective barrier against sexual transmission, since the mid-1990s the proportion of men reporting unsafe sex – including “barebacking”, i.e. unprotected anal intercourse (UAI) in a risk context [7] – seems to have increased [8-11].

UAI is the main HIV transmission route among MSM [12]. Studies designed to increase the understanding about this sexual practice in the different cultures and how it relates to HIV transmission, re-infection and development of HIV antiretroviral resistance may have important public health implications. A meta-analysis from studies conducted in the US estimated an overall UAI prevalence among HIV-infected MSM at 43% (CI 95% 37–48) [12], with lower frequencies when sexual partners were of unknown (30%) or negative (16%) serostatus [12]. These differences may be related to the “sexual harm-reduction” approaches, such as serosorting (the use of the partners’ HIV serostatus - actual or presumed - as a guidance to make decisions when having UAI), strategic positioning (selectively engage in receptive UAI rather than insertive UAI) and negotiated safety (as agreements between steady couples related to sex with casual partners) [13-16].

Data on UAI prevalence and associated factors among Brazilian MSM are scarce, which limits the effectiveness of public policies designed to decrease HIV infection in this population. In a study conducted with over 3,000 HIV-positive and negative Brazilian MSM, UAI prevalence was 36.5%, and the associated factors reported were in accordance with the international literature [17]. However, the authors did not disaggregate data according to HIV serostatus, precluding inferences about attitudes and behaviors associated to HIV transmission.

Behavioral and contextual factors, specifically a higher number of sexual partners [18-20], a history of domestic or sexual violence [21,22], alcohol and illicit drug use, particularly stimulants [23-27], have been associated with UAI among MSM in the international literature. Given the importance of combined anti-retroviral therapy (cART) in the treatment and prevention of HIV infection [28], researchers have also investigated the potential impact of its use, and consequential undetectable viral loads, on UAI reporting. Even though some results showed an increase in UAI among individuals with known undetectable viral load (UVL) [29] or with the belief that UVL decreases transmission [30], most evidence suggest that there is no association between UAI and UVL/cART use [31-34].

Considering the sustained high incidence rate of HIV among MSM and the lack of information on UAI among HIV-infected South American MSM, this paper aims to study the prevalence and associated factors of UAI among HIV-infected MSM in Brazil.

## Methods

A cross-sectional analysis, nested within a cohort study was conducted at Instituto de Pesquisa Clinica Evandro Chagas (IPEC/FIOCRUZ), Rio de Janeiro, Brazil. A convenience sample of HIV–infected and non-infected high risk MSM older than 18 years was enrolled since 2010. Briefly, the cohort was designed to evaluate the prevalence and incidence of anal HPV infection and intraepithelial anal lesions. Participants were considered to be MSM if they had a male sexual partner (s) in the past 12 months, regardless of having a female partner (s).

### Participants

The study population for the present analysis was a subset of HIV-infected MSM enrolled in the parent cohort, who reported having had anal intercourse with men at risk for HIV infection (HIV negative or with an unknown HIV serostatus) within the past 3 months.

**Figure 1:**
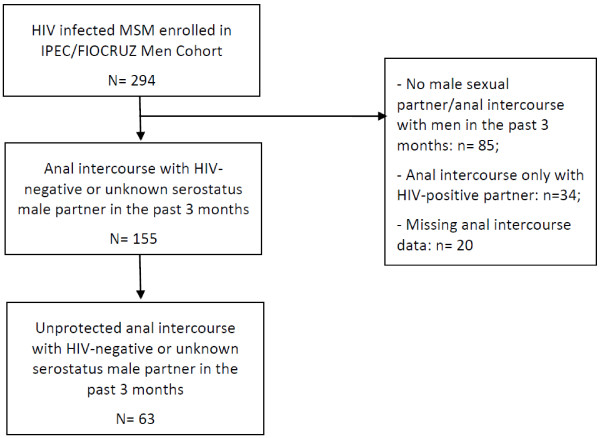
Study population and outcome, IPEC/Fiocruz, 2010–2012.

Two hundred ninety four HIV-infected MSM were enrolled into the IPEC/FIOCRUZ men’s cohort from August 2, 2010 to June 30, 2012. Of these 294 MSM, a total 190 MSM were excluded from this analysis for the following reasons: 85/294 (28.9%) reported no male sexual partner/no anal intercourse in the past 3 months; 34/294 (11.6%) reported anal intercourse exclusively with an HIV-infected male partner; and 20/294 (6.8%) had missing data for anal intercourse data (Figure 1). Final sample was comprised by 155 HIV-infected MSM.

### Measures

The outcome was defined as UAI with at least one man of unknown or negative HIV status in the 3 months preceding the interview.

*Demographic variables* were collected at enrollment visit and included: age, self-reported skin color (white and non-white) and schooling (years of formal education).

*Data on behavior variables were* collected during the enrollment visit via Audio Computer Assisted Interview (ACASI), which included the following:

*Number of male partners* within the last 12 months (“During the past 12 months, how many men and how many transvestite/transsexual/transgender (s) did you have sex with?”);

*Anal intercourse practices in the past 3 months* was measured through the following questions: a-“In the past 3 months, how many HIV-negative male partners did you have insertive anal sex with?”; b-“In the past 3 months, how many male partners, with an unknown HIV-serostatus, did you have insertive anal sex with?”; c- “In the past 3 months, how many HIV-negative male partners did you have receptive anal sex with?”; d-“In the past 3 months, how many male partners, with an unknown HIV-serostatus, did you have receptive anal sex with?”. The questions above were used to select the study population and were also used to formulate the variable “Anal intercourse practices” and its subcategories of *only insertive*, *only receptive* or *both*.

*Unprotected anal intercourse in the past 3 months* was measured by the following questions: a- “In the past 3 months, how many HIV-negative male partners did you have insertive anal sex with, without using condoms?”; b- “In the past 3 months, how many HIV-unknown male partners did you have insertive anal sex with, without using condoms?”; c- “In the past 3 months, how many HIV-negative male partners did you have receptive anal sex with, without using condoms?”; d- “In the past 3 months, how many male partners, with an unknown HIV-serostatus, did you have receptive anal sex with, without using condoms?”.

*Alcohol use, either before or during sex,* within the past 3 months was measured by the question “In the past 3 months, were you drunk or high before or during sex?”);

*Stimulant use, either before or during sex,* within the past 3 months was measured by the question “In the past 3 months, did you use either inhaled or intravenous illicit drugs before or during sex?”; *Lifetime injection drug use* was measured by the question “Have you ever used intravenous illicit drugs in your life?”; however, due to limited observations, this response was not evaluated in the models.

*Commercial sex within the past 3 months* was defined as the exchange of sex for money/other favors and/or the looking for prostitutes: “In the past 3 months, did you have sex for money, drugs or other favors?” and “In the past 3 months, did you look for prostitutes?”.

*A lifetime history of sexual abuse or violence was determined based on a* composite measure of the responses to the following 2 questions: “Did you ever suffer domestic violence in your life?” and “Did you ever suffer sexual abuse in your life?”);

*The Clinical and laboratory variables* included the following:

*Time since HIV diagnosis* was defined as the time since the first HIV-positive serology result until the date of the interview; results were presented using the median (interquartile range).

*Combined antiretroviral therapy (cART)* was defined as the date of issuance of the initial prescription for combined antiretroviral treatment until the date of the interview. Individuals who have a cART prescription were classified as receiving cART.

*CD4*^*+*^*T cell count (cells/mm*^*3*^*) was defined as* the available result closest to the date of the interview. The specimens to measure CD4^+^ T cell count were obtained within a window period of 6 months before and up to 3 months the interview. Data was presented as median and interquartile range.

*HIV viral load (copies/IU).* Specimens were collected on the day of the interview and results were classified as either undetectable (less than 400 copies/IU) or detectable (equal to/higher than 400 copies/IU).

All biological analyses were performed at the IPEC Laboratories, which successfully participates in the College of American Pathologists (CAP) External Quality Assurance (EQA) proficiency testing panels and is certified by the Division of AIDS’ Virology Quality Assurance (VQA) program for quantitative and qualitative HIV assays.

### Statistical analysis

Descriptive analysis and proportions of men who reported UAI in the past 3 months were presented. Chi-square tests and Fisher exact tests were used for categorical variables, and Student *t* test were used for continuous variables. Generalized linear models with logit link and binomial distribution [35] were used to identify independent variables associated with UAI in the past 3 months in the study population. Age (years) and the number of male partners in the last 12 months were modeled as restricted cubic splines with three knots [36].

After univariate analysis, observations with missing information on any selected variable were excluded. Covariates with p values <0.10 were selected, assessed for multi-colinearity using generalized colinearity diagnostics (GVIF) and entered in the initial multivariate model. Based on prior information concerning the effect of age in HIV incidence [37], we forced *a priori* the variable age as a continuous variable into the multivariate models. Covariates with the highest p values in the analysis of deviance (analogous to the likelihood ratio test) were sequentially removed. Variables with statistical significance at 5% (p < 0.05) and those that were not considered a confounder (e.g., when removed, a change equal to or higher than 10% in the odds ratio of any other variable of the model was observed) remained in the final model [38]. The Akaike Information Criterion (AIC) was also used for model selection. The Le Cessie-van Houwelingen-Copas-Hosmer test was used to evaluate the goodness-of-fit of the final model [39]. The predictive ability was evaluated using the Area Under the ROC Curve (AUC). Overdispersion was verified as well as the analysis of the residuals (Pearson and deviance residuals and Leverage or Cook’s distances). The software R 3.0.2 was used to generate all analyses [40].

### Ethics

The study was approved by the IPEC-FIOCRUZ IRB (CAE 0044.0.009.000-09) and all study participants signed an informed consent form prior to enrollment into the cohort.

## Results

One hundred and fifty five MSM who reported anal sex with either an HIV-negative partner or a male partner with an unknown HIV-serostatus within the past 3 months were included. Overall, UAI with an HIV-negative or unknown HIV-serostatus male partner was reported by 40.6% (63/155) of MSM: 7.9% were exclusively with an HIV-negative partner, 76.2% were exclusively with a partner with unknown HIV-serostatus and 15.9% with both, as shown in Table 1.

**Table 1:** Unprotected anal intercourse according to partner (s) HIV-serostatus, IPEC/FIOCRUZ, 2010–2012

**HIV partner serostatus**	**Unprotected anal intercourse within the past 3 months**					
	**No**		**Yes**		**Total**	
	**N**	**%**	**N**	**%**	**N**	**%**
Only HIV-negative	23	25.0	5	7.9	28	18.1
Only unknown HIV	57	62.0	48	76.2	105	67.7
Both	12	13.0	10	15.9	22	14.2
Total	92	100.0	63	100.0	155	100.0

Table 2 describes the sociodemographic, behavioral, clinical and laboratorial characteristics of the study participants by UAI. Median age was 38 years, 53.6% were self-identified as white and 84.2% had more than 8 years of formal education. No significant differences at p < 0.10 level were observed in chance of UAI and skin color (*P* = 0.799) and years of education (*P* = 0.882). The median number of male sexual partners in the 12 months prior to the interview was 6.0; it was higher among those who reported UAI (Median = 10; ***P*** **= 0.038**). Having only a single partner during the 12 months prior to the interview was reported by 12.4%.

**Table 2:** Characteristics of UAI with an HIV negative/unknown serostatus partner within last 3 months (N = 155), IPEC/FIOCRUZ, 2010–2012

**Characteristics**	**Unprotected anal intercourse**			P-value*
	**No**	**Yes**	Total***	
	**92 (%)**	**63 (%)**	**155 (%)**	
Age**	40.5 (31.5-48.0)	37.0 (32.0-43.0)	38 (32.0-45.0)	0.107
White	48 (52.7)	34 (54.8)	82 (53.6)	0.799
Years of education				0.882
< 4	5 (5.6)	2 (3.2)	7 (4.6)	
4 a 8	10 (11.1)	7 (11.3)	17 (11.2)	
> 8	75 (83.3)	53 (85.5)	128 (84.2)	
No of male partners last 12 months**	5.0 (2.0-10.0)	10.0 (3.0-30.0)	6.0 (3.0-15.0)	0.038
Lifetime sexual abuse or violence	23 (25.0)	32 (52.5)	55 (35,9)	0.001
Alcohol use before sex last 3 months	31 (33.7)	26 (41.3)	57 (36,8)	0.337
Stimulant use before sex last 3 months	14 (15.2)	17 (27.0)	31 (20.0)	0.072
Commercial sex last 3 months	34 (37.0)	41 (65.1)	75 (48.4)	0.001
Anal intercourse with men last 3 months				0.002
Only insertive	14 (15.2)	11 (17.5)	25 (16.1)	
Only receptive	33 (35.9)	7 (11.1)	40 (25.8)	
Both	45 (48.9)	45 (71.4)	90 (58,1)	
Months since HIV diagnosis**	70.7 (33.3-162.4)	75.0 (44.1-134.5)	72.0 (33.8-148.8)	0.866
Receiving cART	80 (87.0)	46 (74.2)	126 (81.8)	0.044
CD4 count (cels/mm^3^)**	541.5 (381.0-759.0)	647.0 (414.0-927.0)	581.0 (393.0-837.0)	0.116
Undetectable viral load	47 (51.6)	28 (50.0)	75 (51.0)	0.846

*Chi-square test or Fisher’s exact test were applied for categorical variables. Student *t* test was used for continuous variables.

**Median (IQR).

***Proportions were calculated over the valid number of the cases: Receiving cART (n = 154); White, No of male partners last 12 months, Lifetime sexual abuse or violence, Stimulant use before sex last 3 months (n = 153); Years of education (n = 152); Months since HIV diagnosis (n = 146); CD4 count (n = 143).

Lifetime sexual abuse or domestic violence was reported by 35.9% and was significantly greater among MSM who reported UAI compared to those who did not (p = 0.001). Although 36.8% of MSM reported to be “high” from alcohol use before/during sex in the past 3 months, this was not statistically different from those who reported UAI and those who did not report UAI (*P* = 0.337). Stimulant usage before/during sex in the past 3 months was reported by 20.0% of the MSM, being slightly higher among those who reported UAI when compared to the group who did not. (27.0% vs. 15.2%; *P* = 0.072). Only 4 study participants (2.6%) reported injecting drug use during their lifetime.

Commercial sex in the past 3 months was frequent among all MSM (48.4%) and positively associated with UAI (***P*** **= 0.001**). 58.1% participants reported both insertive and receptive sexual practices, while an exclusively insertive sexual practice was reported only by 16.1%. UAI was less frequently reported in exclusively receptive MSM (11.1%) when compared to those with exclusively insertive (17.5%) or with both insertive and receptive sexual practices (71.4%). (***P*** **= 0.002**).

The median time since HIV diagnosis was 72.0 months and this was not associated with UAI (*P* = 0.866). Almost 82% of MSM were receiving cART at the time of the interview; this was associated with a lower likelihood of UAI (***P*** **= 0.044**). Median CD4 count was higher among MSM who had UAI compared to those who did not report UAI, although no significance was observed at 10% (*P* = 0.116). Roughly half of the participants had an undetectable HIV viral load; this was not associated to a higher likelihood of having UAI (*P* = 0.846).

**Figure 2:**
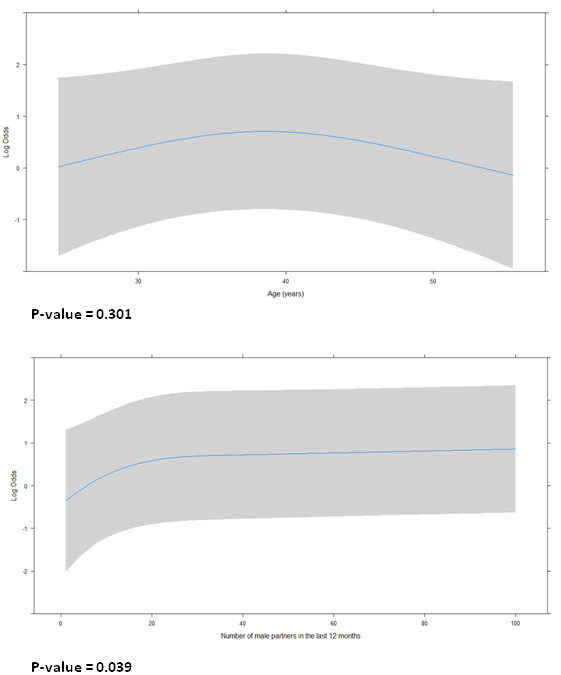
Restricted cubic spline analysis of the functional form of the association between age (years) and number of male partners in the last 12 months and the unprotected anal intercourse in the final model, IPEC/Fiocruz, 2010–2012.

Multi-colinearity was not observed among significant variables (P < 0.10) and age after univariate analysis, which were entered in the initial multivariate model. In the final multivariate model (Table 3 and Figure 2), reporting sexual abuse or domestic violence (OR = 2.70; 95% CI: 1.08-7.01) and having commercial sex within the past 3 months (OR = 2.28; 95% CI: 1.04- 5.10) were positively associated with UAI, whereas the practice of exclusively receptive anal intercourse (OR = 0.21; 95% CI: 0.06-0.75) was negatively associated with UAI. The shape of the association between the number of male partners in the past 12 months and UAI (modeled as the restricted cubic spline with 3 knots: 1.0, 6.0, 35.0) showed that the chance of UAI for each additional male partner increased quickly among participants who reported up to 20 partners and slowly among those who reported more than 20 (p-value = 0.039). Stimulants use before or during sex and receiving cART remained as confounder variables in the final multivariate model. Despite not having a statistically significant impact on UAI, the shape of association for age (years modeled as the restricted cubic spline with 3 knots: 26.1, 38.5, 51.0) is rather flat, though there is a low chance of UAI at the age extremes (p-value = 0.301). There was no overdispersion in the model and the goodness-of-fit as well as the residual analysis were satisfactory.

**Table 3:** Factors associated with unprotected anal intercourse among MSM who have sex with an HIV negative/unknown serostatus partner within the last 3 months (N = 152)*, IPEC/Fiocruz, 2010-2012** ***

Characteristics* **	**Adjusted OR (CI 95%)**	**P-value**
Lifetime sexual abuse or violence	2.70 (1.08 - 7.01)	0.034
Stimulant use before sex last 3 months	1.48 (0.57 - 3.83)	0.418
Commercial sex last 3 months	2.28 (1.04 - 5.10)	0.040
Anal intercourse last 3 months		
Only insertive	1	
Only receptive	0.21 (0.06 - 0.75)	0.018
Receptive and insertive	0.75 (0.25 - 2.22)	0.603
Receiving cART	0.52 (0.18 - 1.47)	0.218

Le Cessie-van Houwelingen-Copas-Hosmer test (P-value): 0.723; AUC: 0.795.

*Three MSM were excluded from initial multivariate model due to missing data in at least one covariate.

**Age was forced *a priori* the variable age into the multivariate models; Number of male partners, Stimulant use before sex, and currently receiving cART was a confounder and remained in the final multivariate model.

***Age (years) and Number of male partners within the last 12 months were modeled as restricted cubic spline with three knots (coefficients not shown).

## Discussion

Most HIV -infected from our study have reported that they did not know their partner’s serostatus, which is in accordance with prior data from Latin America [41]. The frequency of UAI with either negative or unknown HIV status partners was higher (40%) than that described in a meta-analysis conducted with HIV positive MSM from the U.S. (26%) [12]. Therefore, the risk of transmission, as well as re-infection and other sexually transmitted disease (STD) acquisition is increased among this population. Brazilian efforts on prevention, especially promoting condoms use and providing freely cART, have been very efficient in the control of HIV epidemics among general population [2]. However, they were not enough to control HIV transmission among MSM, a population presenting 10.5% HIV point prevalence [42]. Thus, the UAI prevalence data presented in this study indicate that other effective interventions, such as Post-exposure prophylaxis (PEP) and pre-exposure prophylaxis (PrEP) [43-45], are needed as additional prevention tools to vulnerable MSM.

The lifetime history of violence or sexual abuse increased the chances of UAI by almost 3 times. This outcome is particularly concerning seeing that the frequency of UAI for the study population was 40.6%, with greater than half of the men who had UAI reporting previous violence or sexual abuse. Increasing evidence supports the associations of both childhood sexual abuse and intimate partner violence with sexual risk behavior in MSM. An intervention trial [21] aimed to reduce risk behavior in MSM in six American cities found a 39.7% point prevalence of reported child sexual abuse, which was associated with a 1.24 (95% CI: 1.12 to 1.36) increased chance of UAI. Similarly, a positive association was found between intimate partner violence and UAI, depression, and substance abuse among 814 MSM from Chicago [46]. Less is known on these issues in middle-income countries [47] and there are still methodological problems to be solved regarding definitions and study design [48], but results found here point to an important and under evaluated threat to health among this population.

Commercial sex within the past 3 months also increased the likelihood of UAI in MSM. In our study, this variable included both MSM who received money in exchange for sex and/or looked for sexual partners on the streets. Because commercial sex is illegal in many countries, accurately assessing frequency of commercial sex is difficult. As a result, the research data on the association of commercial sex with UAI may be underreported and its association with UAI may be therefore difficult to measure. In a sample of MSM who paid for sex in Pakistan, almost 60% of them reported that their last intercourse event was unprotected [49]; however, more than 80% of interviewed MSM from India reported to have used condoms during their last paid intercourse with other men [50].

Results are also contradictory on the risk of HIV infection among MSM who exchanged sex for money. A meta-analysis from studies conducted in China found no difference in the HIV infection risk for “money-boys” compared to other MSM [51]. However, in a small recent study, including 463 Chinese MSM from the general population, commercial sex increased the chance of HIV infection by 4 times (95% 1.19-13.69) [52]. Even less is known about commercial sex among HIV-infected MSM, including possible cultural differences related to stigma and discrimination that may play a factor in the ability to negotiate the use of safe sex practices. As a result, additional research is still necessary on this topic.

Most MSM (57%) reported both insertive and receptive anal intercourse during the last 3 months, which is in accordance with the international literature [53,54]. MSM who reported having only receptive anal intercourse reported less UAI compared to those who only had insertive sex. These findings may be related to an increased awareness with one’s own health and an intention to protect themselves from super-infection and STD. These data also raise questions about serosorting and strategic positioning as risk reduction practices among Brazilian MSM. Further studies are necessary to evaluate these practices and risk reduction strategies adopted by HIV-positive MSM and their impact on HIV transmission.

The chance of UAI was associated with the number of male sexual partners within the past 12 months (P = 0.039). However, we could observe that for MSM who had more than 20 male sexual partners, the odds of UAI for each additional partner did not increase as quickly when compared to participants who reported up to 20 partners.

In accordance with the recent literature, viral load and cART use [55,12] were not associated with UAI in this study. In contrast to the available literature, our findings indicated that being a younger age and being “high” from alcohol use or recreational stimulant use before/during sex were not associated with UAI. However, these findings must be interpreted with caution and the lack of statistical significance may be associated with the small size of our study population. Alcohol and stimulant consumption may be used to increase sexual performance [56], but intoxication decreases risk perception of HIV transmission [57]. Studies conducted with HIV-infected MSM have found a positive association between sexual risk behavior and the excessive consumption of alcoholic beverages [58,30].

Lastly, it is important to highlight that subjects who reported UAI also reported a higher number of sexual partners, a higher frequency of lifetime history of violence, and had a higher frequency of alcohol and stimulant use before/during sex than MSM who had no UAI. Different theories have been proposed to explain the clustering of different health problems/risky behaviors within the same populations. Dr. Merrill Singer, for example, has proposed the concept of syndemics [59], meaning different problems act synergistically in the worsening of health. Several authors have studied the concept of internalized homophobia as a predictor of risky behavior and psychological issues among MSM [60-62]. These theories are far from disentangling the multiple components related to risky behavior, and there is a debate on their real impact on health [63], but they certainly reinforce the need for a comprehensive approach on treating and preventing HIV among MSM.

As our sample is not probabilistic - and results may not be generalized to a broader population - further research is needed. Nevertheless, our results offer insight on factors associated with unprotected sexual practices among HIV-infected MSM in Brazil. As a limitation, the study was not specifically designed to evaluate intimate partner violence, sexual abuse or other kinds of violence, and as a result the measures were not standardized accordingly. However, given the large prevalence of these factors, those findings indicate a pressing need for studies to support targeted interventions for Brazilian MSM.

## Conclusions

Most MSM included in the present study were not aware of their partner’s HIV serostatus. The high prevalence of UAI with HIV negative or with unknown HIV status male partners indicates that additional prevention strategies are urgently needed for this population. The factors associated with UAI in our study included a lifetime history of violence or sexual abuse, commercial sex and the number of male sexual partners in the past 12 months. The clustering of different health, behavioral, and social problems among these MSM reinforces the need for a comprehensive approach towards treatment and prevention of HIV in this population.

## Competing interests

The authors declare that they have no competing interests.

## Authors’ contributions

CBC participated in the design of the study, performed the statistical analysis and wrote the first draft of the manuscript. All authors contributed to the design of the study, writing and revising the manuscript and all authors approved the final version.

## Pre-publication history

The pre-publication history for this paper can be accessed here:

http://www.biomedcentral.com/1471-2458/14/379/prepub

## References

[B1] Beyrer C Baral SD van Griensven F Goodreau SM Chariyalertsak S Wirtz AL Brookmeyer R Van Griensven F Series HIV in men who have sex with men 1 Global epidemiology of HIV infection in men who have sex with men Lancet 2012 380 367 377 10.1016/S0140-6736(12)60821-6 22819660 PMC3805037

[B2] Ministério da Saude Brasil Boletim Epidemiologico de AIDS/DST 2012 Brasilia: Ministério da Saúde/ Secretaria de Vigilância em Saúde/Departamento de DST, AIDS e Hepatites Virais

[B3] Kerr LRFS Mota RS Kendall C Pinho ADA Mello MB Guimarães MDC Dourado I De Brito AM Benzaken A McFarland W Rutherford G HIV among MSM in a large middle-income country AIDS 2013 27 427 435 10.1097/QAD.0b013e32835ad504 23291540

[B4] Jin F Crawford J Prestage GP Zablotska I Imrie J Kippax SC Kaldor JM Grulich AE Unprotected anal intercourse, risk reduction behaviours, and subsequent HIV infection in a cohort of homosexual men AIDS 2009 23 243 252 10.1097/QAD.0b013e32831fb51a 19098494 PMC2768371

[B5] Moatti J Prudhomme J Coulibaly D Juillet-amari A Akribi HA Msellati P Access to antiretroviral treatment and sexual behaviours of HIV-infected patients aware of their serostatus in Côte d’ Ivoire and the Côte d’ Ivoire Drug Access Initiative Socio-Behavioural Evaluation Group AIDS 2003 33 69 77 10.1097/00002030-200317003-00010 14565612

[B6] Di Pietro N No turning back: HIV and gay male sexuality AIDS Community Res Initiat Am Update 2006 15 12 16

[B7] Bauermeister J Carballo-Dieguez A Ventuneac A Dolezal C Assessing motivations to engage in intentational condomless anal intercourse in HIV-risk contexts (“bareback sex”) among men who have sex with men AIDS Educ Prev 2009 21 156 168 10.1521/aeap.2009.21.2.156 19397437 PMC2699594

[B8] Seng R Rolland M Beck-Wirth G Souala F Deveau C Delfraissy J-F Goujard C Meyer L Trends in unsafe sex and influence of viral load among patients followed since primary HIV infection, 2000–2009 AIDS 2011 25 977 88 10.1097/QAD.0b013e328345ef12 21358375

[B9] Jansen IAV Geskus RB Davidovich U Jurriaans S Coutinho RA Prins M Stolte IG Ongoing HIV-1 transmission among men who have sex with men in Amsterdam: a 25-year prospective cohort study AIDS 2011 25 493 501 10.1097/QAD.0b013e328342fbe9 21192230

[B10] George C Alary M Otis J Demers E Remis RS Mâsse B Lavoie R Vincelette J Parent R Leclerc R Turmel B Nonnegligible increasing temporal trends in unprotected anal intercourse among men who have sexual relations with other men in montreal J Acquir Immune Defic Syndr 2006 42 207 12 10.1097/01.qai.0000200664.24968.4c 16645547

[B11] McFarland W Chen S Weide D Kohn R Klausner J Gay Asian men in San Francisco follow the international trend: increases in rates of unprotected anal intercourse and sexually transmitted diseases, 1999–2002 AIDS Educ Prev 2004 16 13 8 10.1521/aeap.16.1.13.27723 15058707

[B12] Crepaz N Marks G Liau A Mullins MM Aupont LW Marshall KJ Jacobs ED Wolitski RJ Prevalence of unprotected anal intercourse among HIV-diagnosed MSM in the United States: a meta-analysis AIDS 2009 23 1617 29 10.1097/QAD.0b013e32832effae 19584704

[B13] Kippax S Noble J Prestage G Crawford JM Campbell D Baxter D Cooper D Sexual negotiation in the AIDS era: negotiated safety revisited AIDS 1997 11 191 7 10.1097/00002030-199702000-00009 9030366

[B14] Parsons JT Schrimshaw EW Wolitski RJ Halkitis PN Purcell DW Hoff CC Gómez C a Sexual harm reduction practices of HIV-seropositive gay and bisexual men: serosorting, strategic positioning, and withdrawal before ejaculation AIDS 2005 19 Suppl 1 S13 25 15838191 10.1097/01.aids.0000167348.15750.9a

[B15] Xia Q Molitor F Osmond DH Tholandi M Pollack LM Ruiz JD Catania JA Knowledge of sexual partner’ s HIV serostatus and serosorting practices in a California population-based sample of men who have sex with men AIDS 2006 20 April 2081 2089 17053354 10.1097/01.aids.0000247566.57762.b2

[B16] Frost DM Stirratt MJ Ouellette SC Understanding why gay men seek HIV-seroconcordant partners: intimacy and risk reduction motivations Cult Health Sex 2008 10 513 27 10.1080/13691050801905631 18568873

[B17] Rocha GM Kerr LRFS de Brito AM Dourado I Guimarães MDC Unprotected receptive anal intercourse among men who have sex with men in Brazil AIDS Behav 2013 17 4 1288 95 10.1007/s10461-012-0398-4 23325375

[B18] Rosenberg ES Sullivan PS Dinenno EA Salazar LF Sanchez TH Number of casual male sexual partners and associated factors among men who have sex with men: Results from the National HIV Behavioral Surveillance system BMC Public Health 2011 11 189 10.1186/1471-2458-11-189 21439069 PMC3078881

[B19] Zekan S Novotny TE Begovac J Unsafe sexual behavior among HIV-infected patients in Croatia, 2006: prevalence and associated factors AIDS Behav 2008 12 4 Suppl S86 92 18543093 10.1007/s10461-008-9420-2 PMC2715159

[B20] Golin C Marks G Wright J Gerkovich M Tien H-C Patel SN Gardner L O’Daniels C Wilson TE Thrun M Thompson M Raffanti S Quinlivan EB Psychosocial characteristics and sexual behaviors of people in care for HIV infection: an examination of men who have sex with men, heterosexual men and women AIDS Behav 2009 13 1129 42 10.1007/s10461-009-9613-3 19763810 PMC3782535

[B21] Mimiaga M Noonan E Donnel D Safren A Koenen KC Gortmaker S Cleirigh CO Mayer K Childhood sexual abuse is highly associated with HIV-risk taking behavior and infection amomg MSM in the EXPLORE study J Acquir Immune Defic Syndr 2012 51 340 348 19367173 10.1097/QAI.0b013e3181a24b38 PMC3292283

[B22] Pantalone DW Rood BA Morris BW Simoni JM A systematic review of the frequency and correlates of partner abuse in HIV-infected women and men who partner with men J Assoc Nurses AIDS Care 2014 25 1 Suppl S15 35 24070646 10.1016/j.jana.2013.04.003 PMC3875616

[B23] Koblin BA Husnik MJ Colfax G Huang Y Madison M Mayer K Barresi PJ Thomas J Chesney MA Buchbinder S Risk factors for HIV infection among men who have sex with men AIDS 2006 20 September 2005 731 39 16514304 10.1097/01.aids.0000216374.61442.55

[B24] Bajunirwe F Bangsberg DR Sethi AK Alcohol use and HIV serostatus of partner predict high-risk sexual behavior among patients receiving antiretroviral therapy in South Western Uganda BMC Public Health 2013 13 430 10.1186/1471-2458-13-430 23641795 PMC3645971

[B25] Carey JW Mejia R Bingham T Ciesielski C Gelaude D Herbst JH Sinunu M Sey E Prachand N Jenkins RA Stall R Drug use, high-risk sex behaviors, and increased risk for recent HIV infection among men who have sex with men in Chicago and Los Angeles AIDS Behav 2009 13 1084 96 10.1007/s10461-008-9403-3 18498049

[B26] Deiss RG Clark JL Konda KA Leon SR Klausner JD Caceres CF Coates TJ Problem Drinking is Associated With Increased Prevalence of Sexual Risk Behaviors Among men who Have sex With men (MSM) in Lima 2013 Drug Alcohol Depend: Peru 10.1016/j.drugalcdep.2013.01.011 PMC3932358 23434130

[B27] Vosburgh HW Mansergh G Sullivan PS Purcell DW A review of the literature on event-level substance use and sexual risk behavior among men who have sex with men AIDS Behav 2012 16 1394 410 10.1007/s10461-011-0131-8 22323004

[B28] Cohen MS Chen YQ McCauley M Gamble T Hosseinipour MC Kumarasamy N Hakim JG Kumweda J Grinsztejn B Pilotto JH Godbole SV Mehendale S Chariyalertsak S Santos BR Mayer KH Hoffman IF Eshleman SH Piwowar-Manning E Wang L Makhema J Mills LA De Bruyn G Sanne I Eron J Gallant J Havlir D Swindells S Ribaudo H Elharrar V Burns D Prevention of HIV-1 infection with early antiretroviral therapy N Engl J Med 2011 365 6 493 505 10.1056/NEJMoa1105243 21767103 PMC3200068

[B29] Van de Ven P Mao L Fogarty A Rawstorne P Crawford J Prestage G Grulich A Kaldor J Kippax S Undetectable viral load is associated with sexual risk taking in HIV serodiscordant gay couples in Sydney AIDS 2005 19 179 84 10.1097/00002030-200501280-00010 15668543

[B30] Bruce D Kahana S Harper GW Fernández MI The Atn Alcohol use predicts sexual risk behavior with HIV-negative or partners of unknown status among young HIV-positive men who have sex with men AIDS Care 2013 25 559 65 10.1080/09540121.2012.720363 22971018 PMC3522779

[B31] Mayer K Wang L Hoffman I McCauley A Li X Safren S Gamble T Talley J Cottle L Piwowar-Manning E Akelo V Badal-Faesen S Chotirosniramit N Fernandes N Kumarasamy N Shay S Makhema J Panchia B Pilotto J Santos B Cohen M IAS Sustained Treatment as Prevention: Continued Decreases in Unprotected sex and Increases in Virological Suppression After HAART Initiation Among Participants in HPTN 052 XIX International AIDSConference 2012 Washington, D.C: Available at http://www.hptn.org/web%20documents/hptn052/AIDS2012/052MayerMOPDC0106.pdf

[B32] Crepaz N Hart TA Marks G Highly active antiretroviral therapy and sexual risk behavior: a meta-analytic review JAMA 2004 292 224 36 10.1001/jama.292.2.224 15249572

[B33] Phillips AN Cambiano V Nakagawa F Brown AE Lampe F Rodger A Miners A Elford J Hart G Johnson AM Lundgren J Delpech VC Increased HIV incidence in men who have sex with men despite high levels of ART-induced viral suppression: analysis of an extensively documented epidemic PLoS One 2013 8 e55312 10.1371/journal.pone.0055312 23457467 PMC3574102

[B34] Elford J Changing patterns of sexual behaviour in the era of highly active antiretroviral therapy Curr Opin Infect Dis 2006 19 26 32 10.1097/01.qco.0000199018.50451.e1 16374214

[B35] McCullagh P Nelder J Generalized Linear Models 1989 2 London: Chapman & Hall

[B36] Harrell FEJ Lee KL Pollock BG Regression models in clinical studies: determining relationships between predictors and response J Natl Cancer Inst 1988 80 1198 202 10.1093/jnci/80.15.1198 3047407

[B37] Ackers M-L Greenberg AE Lin CY Bartholow BN Goodman AH Longhi M Gurwith M High and persistent HIV seroincidence in men who have sex with men across 47 U.S. cities PLoS One 2012 7 e34972 10.1371/journal.pone.0034972 22529964 PMC3329535

[B38] Greenland S Modeling and variable selection in epidemiologic analysis Am J Public Heal 1989 79 240 9 10.2105/ajph.79.3.340 PMC1349563 2916724

[B39] Hosmer DW Hosmer T Lemeshow S le Cessie S A comparison of goodness-of-fit tests for the logistic regression model Stat Med 1997 16 965 80 10.1002/(SICI)1097-0258(19970515)16:9<965::AID-SIM509>3.0.CO;2-O 9160492

[B40] R Foundation for Statistical Computing: *R* 2013 http://www.r-project.org/

[B41] Nagaraj S Segura ER Peinado J Konda KA Segura P Casapia M Ortiz A Montano SM Clark JL Sanchez J Lama JR A cross-sectional study of knowledge of sex partner serostatus among high-risk Peruvian men who have sex with men and transgender women: implications for HIV prevention BMC Public Health 2013 13 181 10.1186/1471-2458-13-181 23448153 PMC3599550

[B42] De Boni RB Veloso V Grinsztejn B The epidemiology of HIV in Latin America and the Caribbean Curr Opin HIV AIDS 2014 9 2 192 8 10.1097/COH.0000000000000031 24356327

[B43] Grant RM Lama JR Anderson PL McMahan V Liu AY Vargas L Goicochea P Casapía M Guanira-Carranza JV Ramirez-Cardich ME Montoya-Herrera O Fernández T Veloso VG Buchbinder SP Chariyalertsak S Schechter M Bekker L-G Mayer KH Kallás EG Amico KR Mulligan K Bushman LR Hance RJ Ganoza C Defechereux P Postle B Wang F McConnell JJ Zheng J-H Lee J Preexposure chemoprophylaxis for HIV prevention in men who have sex with men N Engl J Med 2010 363 2587 99 10.1056/NEJMoa1011205 21091279 PMC3079639

[B44] Gomez GB Borquez A Caceres CF Segura ER Grant RM Garnett GP Hallett TB The potential impact of pre-exposure prophylaxis for HIV prevention among men who have sex with men and transwomen in Lima, Peru: a mathematical modelling study PLoS Med 2012 9 e1001323 10.1371/journal.pmed.1001323 23055836 PMC3467261

[B45] Schackman BR Eggman AA Cost-effectiveness of pre-exposure prophylaxis for HIV: a review Curr Opin HIV AIDS 2012 7 587 92 10.1097/COH.0b013e3283582c8b 23076124

[B46] Houston E McKirnan DJ Intimate partner abuse among gay and bisexual men: risk correlates and health outcomes J Urban Health 2007 84 681 90 10.1007/s11524-007-9188-0 17610158 PMC2231846

[B47] Finneran C Chard A Sineath C Sullivan P Stephenson R Intimate partner violence and social pressure among gay men in six countries West J Emerg Med 2012 13 260 71 10.5811/westjem.2012.3.11779 22900124 PMC3415831

[B48] Finneran C Stephenson R Intimate partner violence among men who have sex with men: a systematic review Trauma Violence Abuse 2013 14 168 85 10.1177/1524838012470034 23271429 PMC4046894

[B49] Siddiqui AUR Qian H-Z Altaf A Cassell H Shah SA Vermund SH Condom use during commercial sex among clients of Hijra sex workers in Karachi, Pakistan (cross-sectional study) BMJ Open 2011 1 e000154 22021875 10.1136/bmjopen-2011-000154 PMC3191590

[B50] Brahmam GNV Kodavalla V Rajkumar H Rachakulla HK Kallam S Myakala SP Paranjape RS Gupte MD Ramakrishnan L Kohli A Ramesh BM Sexual practices, HIV and sexually transmitted infections among self-identified men who have sex with men in four high HIV prevalence states of India AIDS 2008 22 Suppl 5 S45 57 10.1097/01.aids.0000343763.54831.15 19098479

[B51] Chow EPF Iu KI Fu X Wilson DP Zhang L HIV and sexually transmissible infections among money boys in China: a data synthesis and meta-analysis PLoS One 2012 7 e48025 10.1371/journal.pone.0048025 23209551 PMC3510224

[B52] Zhang L Zhang D Yu B Wang S Liu Y Wang J Li X Shang X Li H Prevalence of HIV infection and associated risk factors among men who have sex with men (MSM) in Harbin, P. R. China PLoS One 2013 8 e58440 10.1371/journal.pone.0058440 23516481 PMC3596395

[B53] McDaid LM Hart GJ Serosorting and strategic positioning during unprotected anal intercourse: are risk reduction strategies being employed by gay and bisexual men in Scotland? Sex Transm Dis 2012 39 735 8 10.1097/OLQ.0b013e31825a3a3c 22902673

[B54] Clark J Salvatierra J Segura E Salazar X Konda K Perez-Brumer A Hall E Klausner J Caceres C Coates T Moderno love: sexual role-based identities and HIV/STI prevention among men who have sex with men in Lima, Peru AIDS Behav 2013 17 1313 28 10.1007/s10461-012-0210-5 22614747 PMC3494756

[B55] Durham MD Buchacz K Richardson J Yang D Wood K Yangco B Brooks JT Sexual risk behavior and viremia among men who have sex with men in the HIV Outpatient Study (HOPS), USA, 2007–2010 J Acquir Immune Defic Syndr 2013 63 372 78 10.1097/QAI.0b013e31828c20d8 23422850 PMC10132175

[B56] Stoner SA George WH Peters LM Norris J Liquid courage: alcohol fosters risky sexual decision-making in individuals with sexual fears AIDS Behav 2007 11 227 37 10.1007/s10461-006-9137-z 16802196

[B57] Fritz K Morojele N Kalichman S Alcohol: the forgotten drug in HIV/AIDS Lancet 2010 376 398 400 10.1016/S0140-6736(10)60884-7 20650516 PMC3015091

[B58] Sander PM Cole SR Stall RD Jacobson LP Eron JJ Napravnik S Gaynes BN Johnson-Hill LM Bolan RK Ostrow DG Joint effects of alcohol consumption and high-risk sexual behavior on HIV seroconversion among men who have sex with men AIDS 2013 27 815 23 10.1097/QAD.0b013e32835cff4b 23719351 PMC3746520

[B59] Singer M Clair S Syndemics and public health: reconceptualizing disease in bio-social context Med Anthropol Q 2003 17 423 41 10.1525/maq.2003.17.4.423 14716917

[B60] Wagner G Brondolo E Rabkin J Internalized homophobia in a sample of HIV + gay men, and its relationship to psychological distress, coping, and illness progression J Homosex 1996 32 91 106 9010828 10.1300/j082v32n02_06

[B61] Ross MW Kajubi P Mandel JS McFarland W Raymond HF Internalized homonegativity/homophobia is associated with HIV-risk behaviours among Ugandan gay and bisexual men Int J STD AIDS 2013 24 409 13 10.1177/0956462412472793 23970711

[B62] Williamson IR Internalized homophobia and health issues affecting lesbians and gay men Health Educ Res 2000 15 97 107 10.1093/her/15.1.97 10788206

[B63] Newcomb ME Mustanski B Moderators of the relationship between internalized homophobia and risky sexual behavior in men who have sex with men: a meta-analysis Arch Sex Behav 2011 40 189 99 10.1007/s10508-009-9573-8 19888643

